# Cheminformatics and computational chemistry in lead optimisation

**DOI:** 10.1186/1758-2946-3-S1-O5

**Published:** 2011-04-19

**Authors:** Andrew R Leach

**Affiliations:** 1 GlaxoSmithKline Research & Development, Stevenage, Hertfordshire SG1 2NY, UK

## 

Lead Optimisation is the process by which a chemical lead series is improved with regard to multiple properties. These properties will vary from project to project but typically include affinity at the target protein, ADME properties and certain toxicological end-points. Techniques from computational chemistry and cheminformatics have a key role to play in many aspects of lead optimisation. A number of methodologies in use at GSK to assist our lead optimisation projects will be described in this talk. The integration of data from multiple data sources is an important starting point, leading to the use of novel computational tools for the analysis and visualisation of structure-activity relationships. The large number of lead optimisation programmes run at GSK over many years has resulted in many thousands of data points. A key question is, what knowledge can be extracted from such large data sets that can help in future optimisation efforts. Here we will summarise some of our work to derive medicinal chemistry "rules of thumb" and to probe for possible isosteric replacements. Finally, the use of experimental design techniques in array chemistry will be discussed.

